# Sexual intercourse, age of initiation and contraception among adolescents in Ireland: findings from the Health Behaviour in School-aged Children (HBSC) Ireland study

**DOI:** 10.1186/s12889-018-5217-z

**Published:** 2018-03-16

**Authors:** Honor Young, Lorraine Burke, Saoirse Nic Gabhainn

**Affiliations:** 1 DECIPHer, Cardiff University, Cardiff, CF10 3BD UK; 2 Discipline of Health Promotion, School of Health Sciences, National University of Ireland, Galway, Ireland

**Keywords:** Sexual behaviour, Ireland, Young people, Contraception, Sexual initiation, Early sex

## Abstract

**Background:**

The need to tackle sexual health problems and promote positive sexual health has been acknowledged in Irish health policy. Young people’s sexual behaviour however remains under-researched with limited national data available.

**Methods:**

This study presents the first nationally representative and internationally comparable data on young people’s sexual health behaviours in Ireland. Self-complete questionnaire data were collected from 4494 schoolchildren aged 15–18 years as part of a broader examination of health behaviour and their context. The prevalence of sexual initiation, very early sexual initiation (< 14 years) and non-condom use at last intercourse are reported and used as outcomes in separate multilevel logistic regression models examining associations between sociodemographic characteristics, lifestyle characteristics and young people’s sexual behaviours.

**Results:**

Overall, 25.7% of boys and 21.2% of girls were sexually initiated. Older age was consistently predictive of initiation for both boys and girls, as were alcohol, tobacco and cannabis involvement, living in poorer neighbourhoods and having good communication with friends. Involvement in music and drama was protective. Very early sexual initiation (< 14 years) was reported by 22.8% of sexually initiated boys and 13.4% of sexually initiated girls, and was consistently associated with rural living, cannabis involvement and bullying others for both. Boys’ very early initiation was predicted by alcohol involvement, receiving unhealthy food from parents and taking medication for psychological symptoms, whereas better communication with friends and more experience of negative health symptoms were protective. Girls’ very early initiation was predicted by being bullied and belonging to a non-Traveller community, whereas taking medication for physical symptoms and attending regular health checks was protective. Condom use was reported by 80% of sexually initiated students at last intercourse. Boys’ condom use was associated with older age, higher family affluence, bullying others, more frequent physical activity and health protective behaviours. For girls, condom use was predicted by belonging to a non-Traveller community, healthy food consumption, higher quality of life and being bullied, whereas taking medication for physical and psychological symptoms was associated with non-condom use.

**Conclusions:**

These nationally representative research findings highlight the importance of focusing on young people as a distinct population subgroup with unique influences on their sexual health requiring targeted interventions and policy.

**Electronic supplementary material:**

The online version of this article (10.1186/s12889-018-5217-z) contains supplementary material, which is available to authorized users.

## Background

Sexual health plays an important role in physical and social well-being. Sexuality is a vital element of the human character. The World Health Organization (WHO) claims that positive sexual health not only influences a person’s physical and mental well-being but is also essential to achieving sustainable development and the realisation of global health and human rights [1]. The views people hold about their sexuality, the behaviours they participate in and the attitudes and values they hold are all shaped by the context in which they live [2]. Contextual factors outside the control of an individual, such as age, disability, sexual orientation, culture and place of residence can also influence the decisions people make [3].

Young people typically engage in experimental behaviours, including sexual behaviours, which help them to establish their individual identity as they make the transition into adulthood [4]. The onset of sexual intercourse is a time of social and personal significance which also has major health implications [5]. A large number of young people in Ireland, and internationally, report sexual initiation during adolescence [6–8]. At this time, norms of behaviour, sexual activity and practices are established [2]. Young people aged 15–24 years who are sexually active are at a greater risk of experiencing adverse health and social outcomes including unplanned pregnancy or Sexually Transmitted Infection (STI) acquisition than older populations [9].

Early sexual initiation (i.e., sexual initiation before 16 years old) has been associated with adverse sexual health outcomes at the time of first intercourse and also later in life [10]. It has implications for adolescents’ self-perception, well-being, social status and later sexual health behaviours [11, 12]. In terms of physiological readiness, girls aged 14 and younger are almost universally too young to have intercourse [13]. Adolescents may not be equipped to manage the psychological consequences of sexual activity; experiencing regret and having a higher risk of depression [14, 15].

Very early sexual initiation (i.e., initiation before 14 years old) has also been associated with sexual intercourse with high risk partners (e.g., intravenous drug users and HIV positive men), multiple sexual partners, STI transmission and sexual and physical violence [16, 17]. The potential risks associated with adolescent sexual behaviour can also be linked to the behavioural and emotional characteristics of their developmental phase. Previous research on the sexual knowledge, attitudes and practices of boys and girls aged 14 and younger tend to report insufficient cognitive preparedness for consensual and safe sexual intercourse [18, 19]. Adolescent sexual behaviour may also be influenced by the under developed decision-making skills associated with this life stage [20]. Studies with adults in Ireland have demonstrated that those who initiated sex earlier were less likely to have used contraception or to have planned intercourse and more likely to regret the timing of first intercourse [21, 22].

Engaging in poorly or unprotected sexual intercourse increases the risk of STIs and unplanned pregnancy [23]. Sexual behaviours such as early sexual initiation (i.e., sexual initiation before 16 years old), inconsistent condom use, multiple sexual partners and ‘casual’ sex are recognised risk factors for unplanned pregnancy and STI transmission [21, 22, 24, 25].

Although a large body of literature suggests that early sexual initiation is associated with adverse outcomes, other research suggests no relationship between early intercourse and depressive symptoms [26] or other problem behaviours [27] and only a modest relationship between adverse outcomes such as lower school attachment [28]. Other findings indicate that the association may be better explained by familial [29] or genetic and environmental factors [30, 31]. Haase et al. [32] identified that not only early, but late timing of first sexual experiences, is associated with lower psychosocial adjustment in selected domains in young adulthood. Contrasting findings highlight the gap that remains in our understanding of the extent of the impact and the mechanisms between early sexual behaviour and associated outcomes.

At present there are limited data on young people’s sexual health and behaviours in Ireland. Data have been collected on the sexual health of young adults, including retrospective accounts of teenage behaviour [6, 21, 22, 33]. Some regional and local studies have also been conducted with young people [7, 34]. Nationally representative sexual health and behaviour data have never before been collected directly from children in Ireland. Consequently there is also limited evidence relating to the prevalence of and associated risk and protective factors for young people’s sexual health and behaviour. In order to gain a more comprehensive understanding of these issues, an approach is required that includes exploring interrelated contextual factors that influence young people’s lives, and is not limited only to individualistic factors [35, 36]. Four sexual behaviour questions have been mandatory for 15-year-old participants in the HBSC study since 2002, however for practical, political and ethical reasons these questions have only been included as mandatory in the Irish study since 2010. Studying sexual behaviour in Ireland is of particular importance given that the legal age of consent for intercourse is 17 years old. A predominantly Catholic country, abortion is not legal in Ireland [37] and access to contraception can be difficult for young people, especially those under the age of consent. The sexual education curriculum, entitled ‘Relationships and Sexuality Education’ or RSE is delivered in the context of the broader ‘Social and Personal Health Education’ (SPHE). Prompted by concern around adolescent pregnancy in the mid 1990s, it takes a holistic perspective with key objectives focused on relationships and decision-making. Schools and educational stakeholders, including parents, have a major role in determining the approach taken within schools. Each school is mandated to develop its own RSE policy which determines what is taught, and how, in line with its usually Roman Catholic ethos and to do so in collaboration with parents. There are no strict curricular criteria or resources employed across all schools. This can translate into a focus on relationships education and marriage preparation, rather than sexuality education or sexual health promotion [38].

This paper provides the first nationally representative data on young people’s sexual health and behaviour. It provides a novel contribution to the understanding of young people’s sexual behaviour across Ireland. It explores the prevalence of sexual health behaviours and incorporates an approach to explore the individual and social risk and protective sociodemographic and lifestyle behaviours associated with young people’s sexual behaviours. The paper uses data from the 2010 Health Behaviour in School-aged Children (HBSC) international study and explores the following research questions;

1) What are the prevalence rates of sexual initiation, age of sexual initiation, and contraceptive use at last intercourse of young people aged 15–18 years old in Ireland;

2) What are the socio-demographic and lifestyle characteristics related to 15–18 year old young people’s experience of sexual initiation, age of initiation, and contraceptive use at last intercourse.

## Methods

### Data

The Health Behaviour in School-aged Children (HBSC) study is a WHO cross-national research project that aims to increase the understanding of young people’s health, well-being and behaviours, including sexual behaviours. Internationally comparable data are collected from over 200,000 students aged 11, 13 and 15 year olds every four years across 43 participating countries [39]. Data collection in Ireland is extended to include young people aged 9 to 18 years. The study uses an anonymous self-completion international standardised questionnaire; administered in classrooms to gain the perspectives of a representative proportion of 9–18 year old school-going children in the Republic of Ireland. The findings are used to inform and influence population health, health services and health education policy and practice at local, national and international levels.

### Sample

In 2010 data were collected from 256 schools in Ireland totalling 16,060 children of whom 4494 were aged 15–18 years and used in the current analysis [40]. Sampling for the HBSC Ireland 2010 study was conducted to reflect a representative proportion of young people in Ireland. Census data were used to indicate population distribution across geographic regions. The sampling frame consisted of both primary and secondary schools in Ireland; lists of which were provided by the Department of Education and Skills. Schools within geographical regions were randomly selected for participation, followed by the random selection of classes within schools. A total of 256 schools were recruited with a response rate of 67%. Overall, 16,060 school children participated in the HBSC 2010 survey with a response rate of 85%. The primary sampling unit for data collection in the HBSC is at classroom level. Classroom clustering was accounted for using the Complex Samples function on SPSS. The data is not spread equally over 15–18 year old students. The sample size for 18 year olds is the smallest and is not likely to be representative of 18 year olds in the population. This group however represents 18 year olds who are still attending secondary education.

### Ethics

Ethical approval was granted by The National University of Ireland, Galway Research Ethics Committee. In accordance with the HBSC international protocol, sexual behaviour items were asked only of students aged 15 years and older as the majority of younger people are less likely to have experienced sexual intercourse and to avoid potential objection from parents and school management teams.

### Measures

All survey questions were designed by members of the HBSC international study network or adapted from existing measures [39]. All components are subjected to agreed piloting and pre-testing protocols and peer-reviewed by subject experts. The sexual health questions reported on here were discussed with children and assessed by them in advance of their use [41–43].

#### Sociodemographics

Socio-demographic questions were based on the 2010 HBSC international protocol [39]. Gender was measured by asking participants whether they are a boy or a girl. Age was measured by asking participants the month and year in which they were born, and calculated at the time of questionnaire completion. Social class was measured by asking participants the occupations of their parents. The Irish Central Statistics Office [44] measure of social classification (occupation and income level of respondent) was used to identify social class for each respondent. Social class is represented by SC 1–2, SC 3–4 and SC 5–6, corresponding to high, middle and low social classes respectively. Responses were taken as the highest social class available for either mother or father. The HBSC Family Affluence Scale (FAS) [45] is based on a set of questions about the material conditions of the households in which participants live, including car ownership, bedroom occupancy, holidays and home computers. A composite score is calculated for each participant providing values corresponding to low, middle and high family affluence. Urban/Rural status was measured by asking participants for their residential status; city, town, village or country. Living in town or city represented urban status, while rural status was country or village. Household composition was measured by asking to identify the people who live in the home where they live all or most of the time. Responses were categorised into those who lived with both parents and those who did not. Disability/Chronic illness status was assessed through the question ‘Do you have a long-term illness, disability, or medical condition (like diabetes, asthma, allergy or cerebral palsy) that has been diagnosed by a doctor?’ Response options were ‘yes’ and ‘no,’

#### Lifestyle characteristics

Individual items (*n* = 88) relating to the following four domains were identified from the 2010 HBSC survey [39]; 1) physical activity, 2) negative lifestyle behaviours, 3) health and 4) socio-cultural environment. The items identified from the survey can be found in Additional file 1; full item details, including response options, can be found in Currie et al. [39, 46] The number of items identified inhibited detailed analysis and consequently a Categorical Principle Components Analysis (CatPCA) [47] was conducted on the items to understand their structure and uni-dimensionality, while reducing the dimensionality of the data for the purpose of further analyses. All meaningful explanatory factors were extracted for each domain. The CatPCA identified five factors for positive lifestyle behaviours explaining 66.1% of the variance; five factors for negative lifestyle behaviours explaining 58.2% of the variance; four factors for health explaining 51.5% of the variance and nine factors for socio-cultural environment explaining 52.4% of the variance (See Additional file 1). These factors were entered into the logistic regression analyses.

### Sexual behaviour

#### Sexual initiation

Sexual initiation was measured by asking ‘Have you ever had sexual intercourse? (Sometimes this is called “making love”, “having sex” or “going all the way”).’ No anatomical definition was provided, nor did the question differentiate between consensual or non-consensual sex. Response options included ‘Yes,’ and ‘No.’ The age of sexual initiation was measured by asking ‘How old were you when you had sexual intercourse for the first time?’ Response options included ‘I have never had sexual intercourse,’ ‘11 years or younger,’ ‘12 years old,’ ‘13 years old,’ ‘14 years old,’ ‘15 years old,’ ‘16 years old’ and ‘17 years old and older.’

#### Contraceptive use

In order to measure pregnancy prevention, participants were asked ‘The last time you had sexual intercourse, what method(s) did you or your partner use to prevent pregnancy?’ Response options included; ‘I have never had sexual intercourse,’ ‘no method was used to prevent pregnancy,’ ‘birth control pills,’ ‘condoms,’ ‘withdrawal,’ ‘some other method,’ and ‘not sure.’ Participants were provided with space to report ‘other’ pregnancy prevention methods used at last intercourse. Participants were also asked a separate question on condom use (designed to address STI prevention) by asking ‘The last time you had sexual intercourse, did you or your partner use a condom?’ Response options included ‘I have never had sexual intercourse,’ ‘yes,’ and ‘no.’ Inconsistent responses (e.g., no method of contraception followed by condom use (*n* = 68)) were attributed the highest level of protection provided (e.g., condom use). Corrected figures (e.g., for no method of contraception) are reported in Table 1.

**Table 1:** Prevalence of sociodemographics, sexual initiation, age of initiation and contraceptive use of participants

	Boys (*n* = 2408, 53.6%) % (n)	Girls (*n* = 2071, 46.1%) % (n)
Age	Missing *n* = 0	Missing *n* = 0
15 years	43.8 (1055)	44.3 (918)
16 years	34.9 (841)	36.1 (747)
17 years	18.2 (432)	17.0 (353)
18 years	3.1 (74)	2.6 (53)
Social Class (SES)	Missing *n* = 421	Missing *n* = 280
Low	11.9 (286)	12.7 (264)
Middle	32.7 (787)	32.0 (663)
High	38.0 (914)	41.7 (864)
Family affluence (FAS)	Missing *n* = 274	Missing *n* = 136
Low	9.2 (221)	10.0 (207)
Middle	39.2 (945)	36.5 (756)
High	40.2 (968)	46.9 (972)
Urban/Rural status	Missing n = 0	Missing *n* = 0
Urban	61.2 (1474)	66.8 (1383)
Rural	38.8 (934)	33.2 (688)
Traveller status	Missing *n* = 30	Missing *n* = 35
Traveller	1.6 (38)	1.1 (23)
Non-traveller	97.2 (2340)	97.2 (2013)
Disability/Chronic illness (D/CI)	Missing *n* = 51	Missing *n* = 39
D/CI	21.0 (506)	19.8 (410)
No D/CI	76.9 (1851)	78.3 (1622)
Household composition	Missing *n* = 13	Missing *n* = 13
Living with both parents	74.0 (1781)	74.4 (1541)
Not living with both parents	25.5 (613)	25.0 (517)
Sexual intercourse	Missing *n* = 257	Missing *n* = 148
Yes	25.7 (619)	21.2 (439)
No	63.6 (1532)	71.7 (1484)
Age of initiation	Boys (*n* = 619) % (n) Missing *n* = 47	Girls (*n* = 439) % (n) Missing *n* = 12
11 years or younger	11.1 (69)	4.1 (18)
12 years	3.9 (24)	3.2 (14)
13 years	7.8 (48)	6.2 (27)
14 years	18.3 (113)	20.7 (91)
15 years	27.3 (169)	34.2 (150)
16 years	18.9 (117)	23.0 (101)
17 years or older	5.2 (32)	5.9 (26)
Early sexual initiation	Missing *n* = 196	Missing *n* = 139
Early sexual initiation (ages 14–15)	45.6 (282)	54.9 (241)
Very early sexual initiation (aged < 14)	22.8 (141)	13.4 (59)
Contraception at last intercourse^a^		
Condom	79.0 (489)	80.0 (351)
Pill	19.4 (120)	26.9 (118)
Withdrawal	14.5 (90)	14.6 (64)
No method	10.5 (60)	6.8 (27)

^a^These questions were not mutually exclusive and therefore do not total 100%

### Analysis

Inconsistent or unfeasible responses were recoded (e.g., students who did not report sexual initiation but who reported an age of initiation and/or the use of contraceptive methods at last intercourse were recorded as not initiated) (*n* = 45). Students who reported condom use on either question relating to condom use (pregnancy prevention or STI prevention) were credited with condom use (*n* = 66). Respondents were asked to select all applicable response options for questions relating to contraceptive use at last intercourse. These responses were not mutually exclusive and consequently did not total 100%.

The analysis explores the prevalence of sexual initiation, age of initiation and contraceptive use according to gender and age. Gender differences warranted separate analyses for boys and girls. The associations between sexual initiation, age of initiation and contraceptive use at last intercourse and sociodemographic characteristics (social class, family affluence, rurality, disability status and household composition) were first explored.

Experience of sexual initiation, very early sexual initiation and non-condom use were used as outcomes in separate multilevel logistic regression models to examine the association between sociodemographic characteristics and lifestyle characteristics and young people’s sexual behaviours. Separate analyses were conducted for boys and girls. Significance levels for models relating to very early initiation and non-condom use are reported at *p* < .10 and *p* < .05 because of the small sample size of sexually initiated participants and lack of power. All sociodemographic characteristics were added into multivariate models. Lifestyle characteristics covariates were entered into the regression models based on the CatPCA factors (See Additional file 1). Multi-collinearity was checked prior to conducting multivariate analyses; tolerances were above 0.1 and VIF scores were less than 3. Fully adjusted models are presented (Tables 2, 3 and 4). Analyses were conducted in SPSS Statistics for Windows, Version 21.0 [48]. Classroom clustering was accounted for using the Complex Samples function. No adjustments were made to *p*-value levels to account for multiple comparisons. Listwise deletion of variables was employed.

**Table 2:** Chi^2^ associations between sexual initiation and sociodemographics, and adjusted odds ratios (95% confidence intervals) for the association between sexual initiation and sociodemographic and lifestyle characteristics by gender

	Chi^2^ association between sexual initiation and sociodemographic characteristics				Adjusted odds ratios (95% confidence intervals) for the association between sexual initiation and sociodemographic and lifestyle characteristics			
	Boys (*n* = 2151)		Girls (*n* = 1923)		Boys (*n* = 1742)		Girls (*n* = 1604)	
	Sexually initiated % (n)	Not sexually initiated % (n)	Sexually initiated % (n)	Not sexually initiated % (n)	(EXP B) OR	95% C.I. for EXP (B)	(EXP B) OR	95% C.I. for EXP (B)
Sociodemographics								
Age								
15 years	26.5 (243)***	73.5 (673)	16.2 (136)***	83.8 (703)	1.302**	1.075–1.578	1.628***	1.326–1.998
16 years	25.7 (196)	74.3 (568)	21.9 (155)	78.1 (552)				
17 years	38.4 (155)	61.6 (249)	37.2 (124)	62.8 (209)				
18 years	37.3 (25)	62.7 (42)	54.5 (24)	45.5 (20)				
Social Class (SES)								
Low	29.3 (76)***	70.7 (183)	27.9 (68)***	72.1 (176)	1 (ref)		1 (ref)	
Middle	30.2 (221)	69.8 (512)	25.0 (157)	75.0 (470)	1.019	0.687–1.512	1.109	0.679–1.812
High	23.2 (197)	76.8 (652)	17.6 (145)	82.4 (678)	0.889	0.593–1.333	0.732	0.461–1.162
Family affluence (FAS)								
Low	35.0 (75)	65.0 (139)	29.9 (60)***	70.1 (141)	1.225	0.714–2.100	1.351	0.766–2.382
Middle	27.7 (253)	72.3 (662)	25.2 (185)	74.8 (550)	0.956	0.722–1.265	1.176	0.856–1.615
High	26.8 (249)	73.2 (681)	19.3 (180)	80.7 (754)	1 (ref)		1 (ref)	
Urban/rural status								
Urban	29.4 (389)	70.6 (935)	22.2 (285)	77.8 (1000)	1.001	(0.699–1.433)	0.964	0.671–1.385
Rural	27.8 (230)	72.2 (597)	24.1 (154)	75.9 (484)	1 (ref)		1 (ref)	
Traveller status^b^								
Traveller	84.4 (28)***	15.2 (5)	45.5 (10)**	54.5 (12)	4.554*	1.210–17.140	2.873	0.885–9.321
Non-traveller	27.9 (584)	72.1 (1510)	22.6 (423)	77.4 (1451)	1 (ref)		1 (ref)	
Disability/Chronic illness (D/CI)								
D/CI	30.7 (143)	69.3 (323)	26.8 (105)*	73.2 (287)	1 (ref)		1 (ref)	
No D/CI	28.2 (467)	71.8 (1190)	21.7 (327)	78.3 (1179)	0.873	0.643-1.186	0.901	0.621-1.307
Household composition								
Living with both parents	25.0 (411)***	75.0 (1232)	19.8 (287)***	80.2 (1164)	1 (ref)		1 (ref)	
Not living with both parents	40.7 (203)	59.3 (296)	32.3 (151)	67.7 (317)	1.604**	1.134–2.268	1.233	0.883–1.721
Lifestyle characteristics								
Positive lifestyle behaviours								
Frequency of physical activity					1.127	0.976–1302	1.051	0.897–1.232
Active travel					0.953	0.824–1.102	0.827	0.681–1.004
Eating breakfast					0.958	0.825–1.112	1.070	0.928–1.234
Healthy food consumption					1.021	0.903–1.156	0.865	0.739–1.013
Health protective behaviour					0.927	0.802–1.071	0.972	0.760–1.244
Negative lifestyle behaviours								
Alcohol involvement					2.162***	1.831–2.554	2.098***	1.779–2.474
Cannabis involvement					1.581***	1.374–1.818	1.531***	1.246–1.881
Unhealthy food from parents					1.039	0.911–1.185	1.086	0.937–1.258
Tobacco involvement					1.409***	1.228–1.618	1.833***	1.593–2.110
Unhealthy food consumption					1.114	0.976–1.272	1.202*	1.036–1.394
Health								
Experience of health symptoms					1.105	0.951–1.284	1.138	0.950–1.362
Quality of life					1.034	0.877–1.220	0.952	0.807–1.124
Medication for physical symptoms					1.167*	1.012–1.345	1.117	0.962–1.296
Medication for psychological symptoms					1.093	0.935–1.278	1.068	0.935–1.220
Socio-cultural environment								
Communication with friends					1.307***	1.140–1.497	1.561***	1.238–1.968
Sense of community					1.131	0.984–1.300	1.125	0.952–1.329
Poor neighbourhood environment					1.313***	1.139–1.514	1.211*	1.038–1.413
Bullying others					1.137	0.972–1.330	1.444*	1.092–1.910
Music and drama					0.754**	0.622–0.913	0.869*	0.759–0.994
Being bullied					1.052	0.925–1.197	1.248*	1.052–1.482
Club or team activities					1.267**	1.108–1.449	1.009	0.861–1.183
Parental communication					0.986	0.838–1.160	0.918	0.774–1.088
Health check ups					1.011	0.876–1.166	1.074	0.938–1.230

* *p* ≤ .05; ** *p* ≤ .01; *** *p* ≤ .001. Nagelkerke R^2^ = 0.631 (boys), Nagelkerke R^2^ = 0.399 (girls)

^b^Caution must be taken when extrapolating from this finding given the small sample size

**Table 3:** Chi^2^ associations between early sexual initiation and sociodemographics by gender, and adjusted odds ratios (95% confidence intervals) for the association between very early sexual intercourse and sociodemographic and lifestyle characteristics by gender

	Chi^2^ association between early sexual initiation and sociodemographic characteristics				Adjusted odds ratios (95% confidence intervals) or the association between very early sexual initiation (< 14 years) and sociodemographic and lifestyle characteristics			
	Boys (*n* = 423) % (n)		Girls (*n* = 300) % (n)		Boys (*n* = 307)		Girls (*n* = 237)	
	Very early initiation	Early initiation	Very early initiation	Early initiation	(EXP B) OR	95% C.I. for EXP (B)	(EXP B) OR	95% C.I. for EXP (B)
	(*n* = 141)	(*n* = 282)	(*n* = 59)	(*n* = 241)				
Age								
15 years	42.2 (89)***	57.8 (122)	20.2 (26)*	79.8 (103)	0.774	0.457–1.310	0.819	0.439–1.529
16 years	21.3 (30)	78.8 (111)	11.4 (12)	88.6 (93)				
17 years	29.7 (19)	70.3 (45)	30.4 (17)	69.6 (39)				
18 years	42.9 (3)	57.1 (4)	40.0 (4)	60.0 (6)				
Social Class (SES)								
Low	26.3 (15)	73.7 (42)	21.4 (9)	78.6 (33)	1.456	0.598–3.543	1.885	0.527–6.742
Middle	28.8 (44)	71.2 (109)	17.1 (20)	82.9 (97)	0.985	0.426–2.280	1.144	0.331–3.955
High	36.4 (44)	63.6 (77)	19.8 (18)	80.2 (73)	1(ref)		1(ref)	
Family affluence (FAS)								
Low	34.0 (17)	66.0 (33)	31.0 (13)	69.0 (29)	1(ref)		1(ref)	
Middle	29.5 (49)	70.5 (117)	17.8 (23)	82.2 (106)	0.952	0.489–1.854	1.048	0.408–2.692
High	30.9 (55)	69.1 (123)	17.4 (21)	82.6 (100)	0.775	0.276–2.175	0.812	0.167–3.941
Urban/rural status								
Urban	27.8 (72)*	72.2 (187)	16.0 (31)*	84.0 (163)	1(ref)		1(ref)	
Rural	42.1 (69)	57.9 (95)	26.4 (28)	73.6 (78)	2.640**	1.303–5.347	3.107*	1.061–9.105
Traveller status^b^								
Traveller	68.0 (17)***	32.0 (8)	33.3 (3)	66.7 (6)	1(ref)		1(ref)	
Non-traveller	30.6 (120)	69.4 (272)	19.2 (55)	80.8 (232)	0.418	0.091–1.914	14.565*	1.489–142.450
Disability/Chronic illness (D/CI)								
D/CI	36.2 (34)	62.3 (60)	22.7 (15)	77.3 (51)	1(ref)		1(ref)	
No D/CI	32.2 (104)	67.8 (219)	18.8 (43)	81.2 (186)	0.592	0.286–1.228	0.549	0.177–1.698
Household composition								
Living with both parents	33.2 (89)	66.8 (179)	16.8 (31)	83.2 (153)	1(ref)		1(ref)	
Not living with both parents	33.6 (51)	66.4 (101)	24.3 (28)	75.7 (87)	1.109	0.539–2.280	1.781	0.759–4.183
Lifestyle characteristics								
Positive lifestyle behaviours								
Frequency of physical activity					0.945	0.693–1.288	0.686	0.428–1.101
Active travel					1.151	0.857–1.546	0.882	0.553–1.407
Eating breakfast					0.912	0.689–1.208	0.946	0.580–1.543
Healthy food consumption					0.966	0.703–1.328	1.094	0.703–1.703
Health protective behaviour					0.895	0.711–1.127	0.859	0.548–1.347
Negative lifestyle behaviours								
Alcohol involvement					1.303*	1.040–1.632	1.345	0.882–2.052
Cannabis involvement					1.239^a^	0.998–1.537	1.634**	1.185–2.254
Unhealthy food from parents					1.423*	1.026–1.974	0.867	0.511–1.471
Tobacco involvement					0.930	0.726–1.193	1.321	0.909–1.921
Unhealthy food consumption					1.137	0.826–1.565	1.118	0.741–1.686
Health								
Experience of health symptoms					0.599**	0.416–0.863	0.932	0.647–1.342
Quality of life					1.054	0.766–1.451	1.084	0.683–1.720
Medication for physical symptoms					0.986	0.750–1.297	0.613*	0.414–0.908
Medication for psychological symptoms					1.296*	1.036–1.621	0.935	0.630–1.386
Socio-cultural environment								
Communication with friends					0.709**	0.552–0.911	0.987	0.576–1.692
Sense of community					1.191	0.893–1.588	1.248	0.802–1.943
Poor neighbourhood environment					0.834	0.625–1.111	1.429	0.912–2.238
Bullying others					1.216^a^	0.988–1.496	1.609*	1.110–2.333
Music and drama					1.131	0.743–1.170	0.984	0.623–1.556
Being bullied					1.103	0.835–1.458	1.626*	1.085–2.436
Club or team activities					1.156	0.862–1.551	0.968	0.653–1.434
Parental communication					0.865	0.606–1.234	0.875	0.518–1.479
Health check ups					0.790	0.580–1.076	0.369***	0.234–0.582

^a^≤.1, * *p* ≤ .05; ** *p* ≤ .01; *** *p* ≤ .001. Nagelkerke R^2^ = 0.173 (Boys), Nagelkerke R^2^ = 0.187 (Girls)

^b^Caution must be taken when extrapolating from this finding given the small sample size

**Table 4:** Chi^2^ associations between condom use at last intercrouse and sociodemographics by gender, and adjusted odds ratios (95% confidence intervals) for the association between non-condom use at last intercourse and sociodemographic and lifestyle characteristics by gender

	Chi^2^ association between condom use and sociodemographic characteristics				Adjusted odds ratios (95% confidence intervals) for the association between non-condom use and sociodemographic and lifestyle characteristics			
	Boys (n = 619) % (n)		Girls (n = 439) % (n)		Boys (*n* = 459)		Girls (*n* = 349)	
	Condom use	Non-condom use	Condom use	Non-condom use	(EXP B) OR	95% C.I. for EXP (B)	(EXP B) OR	95% C.I. for EXP (B)
	(*n* = 489)	(*n* = 130)	(*n* = 351)	(*n* = 88)				
Age								
15 years	71.6 (174)***	28.4 (69)	77.2 (105)	22.8 (31)	0.669*	0.466–0.961	0.964	0.686–1.353
16 years	83.2 (163)	16.8 (33)	85.2 (132)	14.8 (23)				
17 years	82.6 (128)	17.4 (27)	77.4 (96)	22.6 (28)				
18 years	96.0 (24)	4.0 (1)	75.0 (5.1)	25.0 (6)				
Social Class (SES)								
Low	85.5 (65)	14.5 (11)	82.4 (56)	17.6 (12)	1.368	0.611–3.063	0.681	0.237–1.960
Middle	78.7 (174)	21.3 (47)	76.4 (120)	23.6 (37)	1.684	0.804–3.527	1.445	0.543–3.846
High	81.7 (161)	18.3 (36)	84.8 (123)	15.2 (22)	1(ref)		1(ref)	
Family affluence (FAS)								
Low	80.0 (60)	20.0 (15)	73.3 (44)	26.7 (16)	1(ref)		1(ref)	
Middle	79.4 (201)	20.6 (52)	82.2 (152)	17.8 (33)	1.224	0.709–2.113	0.836	0.418–1.672
High	81.1 (202)	18.9 (47)	80.0 (340)	20.0 (36)	0.392^a^	0.139–1.100	0.952	0.371–2.441
Urban/rural status								
Urban	81.0 (315)	19.0 (74)	80.0 (228)	20.0 (57)	1(ref)		1(ref)	
Rural	75.7 (174)	24.3 (56)	79.9 (123)	20.1 (31)	1.164	0.606–2.235	0.625	0.302–1.292
Traveller status^b^								
Traveller	71.4 (20)	28.6 (8)	50.0 (5)*	50.0 (5)	1(ref)		1(ref)	
Non-traveller	79.8 (466)	20.2 (118)	80.9 (342)	19.1 (81)	0.709	0.208–2.418	.076*	0.010–0.556
Disability/Chronic illness (D/CI)								
D/CI	74.8 (107)	25.2 (36)	84.8 (89)	15.2 (16)	1(ref)		1(ref)	
No D/CI	80.3 (375)	19.7 (92)	78.6 (257)	21.4 (70)	0.716	0.385–1.333	1.581	0.709–3.526
Household composition								
Living with both parents	80.8 (332)	19.2 (79)	81.2 (233)	18.8 (54)	1(ref)		1(ref)	
Not living with both parents	75.9 (154)	24.1 (49)	77.5 (117)	22.5 (34)	1.517	0.848–2.712	0.895	0.450–1.783
Lifestyle characteristics								
Positive lifestyle behaviours								
Frequency of physical activity					0.700**	0.541–0.907	0.931	0.666–1.302
Active travel					1.022	0.796–1.312	0.722	0.541–1.101
Eating breakfast					0.993	0.752–1.310	1.048	0.754–1.456
Healthy food consumption					0.998	0.774–1.287	0.796^a^	0.612–1.035
Health protective behaviour					0.688***	0.566–0.836	0.796	0.617–1.234
Negative lifestyle behaviours								
Alcohol involvement					1.009	0.809–1.260	1.049	0.751–1.465
Cannabis involvement					0.995	0.834–1.187	1.202	0.921–1.569
Unhealthy food from parents					0.893	0.669–1.191	0.923	0.690–1.234
Tobacco involvement					0.932	0.744–1.168	0.977	0.784–1.217
Unhealthy food consumption					0.918	0.698–1.206	0.848	0.640–1.123
Health								
Experience of health symptoms					1.002	0.716–1.402	1.060	0.792–1.419
Quality of life					0.956	0.701–1.302	0.726^a^	0.522–1.010
Medication for physical symptoms					1.143	0.886–1.475	1.243^a^	0.961–1.608
Medication for psychological symptoms					1.087	0.893–1.324	1.254*	1.025–1.533
Socio-cultural environment								
Communication with friends					0.845	0.659–1.082	0.906	0.630–1.302
Sense of community					1.044	0.817–1.334	1.048	0.769–1.426
Poor neighbourhood environment					1.052	0.847–1.306	0.876	0.620–1.239
Bullying others					0.839^a^	0.698–1.007	0.975	0.703–1.353
Music and drama					0.965	0.674–1.382	1.077	0.812–1.430
Being bullied					1.126	0.886–1.432	0.786^a^	0.590–1.047
Club or team activities					0.965	0.750–1.241	1.019	0.725–1.432
Parental communication					1.075	0.761–1.518	0.875	0.648–1.180
Health check ups					0.925	0.718–1.192	1.074	0.746–1.547

^a^≤.1, *p ≤ .05; ** *p* ≤ .01; *** *p* ≤ .001. Nagelkerke R^2^ = 0.305 (Boys), Nagelkerke R^2^ = 0.415 (Girls)

^b^Caution must be taken when extrapolating from this finding given the small sample size

## Results

Data were collected from 256 schools in Ireland totalling 16,060 children of whom 4494 were aged 15–18 years. Sociodemographic characteristics are detailed in Table 1. Around half the sample were boys (53.6%), and the highest proportion comprised of 15 year olds (43.8%, boys and 44.3%, girls). The smallest proportion were those aged 18 years (3.1%, boys and 2.6%, girls). A total of 11.9% of boys and 12.7% of girls were categorised as low SES, and 9.2% of boys and 10.0% of girls were classed as low FAS. Around two thirds of the sample reported living in urban areas (61.2%, boys and 66.8%, girls), and around one fifth of the sample reported living with a disability of chronic illness. Three quarters of the sample reported living with both parents.

### Sexual initiation

A total of 25.7% of boys and 21.2% of girls reported sexual initiation (Table 1). Table 2 details the Chi^2^ tests of association between sociodemographic characteristics and participants reporting sexual initiation. Older participants were more likely to be sexually initiated than younger participants. Clear social gradients were identified in relation to sexual behaviour; higher proportions of boys and girls from middle and lower SES groups reported sexual initiation compared to boys and girls from higher SES groups. Girls from low FAS groups reported significantly higher sexual initiation compared to those from middle and high FAS groups. A similar pattern was identified for boys but was not statistically significant. There were no differences in reported sexual initiation according to urban or rural residential status. A higher percentage of boys and girls who did not live with both parents reported sexual initiation compared those who lived with both parents. Girls who reported a D/CI reported higher sexual initiation compared to girls’ who reported no D/CI. No association was found among boys. A higher propotion of boys and girls from Traveller communities reported sexual initiation compared to those from non-Traveller communities, however caution must be exercised when extrapolating from this finding due to small sample sizes.

### Predictors of sexual initiation

Table 2 also presents the adjusted odds ratios (95% confidence intervals) for the association between sexual initiation and sociodemographic and lifestyle characteristics among boys and girls. Older boys were more likely to report sexual initiation, as were boys who reported living in poorer neighbourhoods and those from Traveller communities (although caution must be exercised with this finding due to small sample size). Living with both parents reduced the likelihood of boys reporting sexual initiation as did higher involvement in music and drama activities. Boys who reported sexual initiation also reported better communication with their friends and engaging in more extra-curricular activities. Sexual initiation was associated with more involvement with alcohol, tobacco and cannabis in the last 30 days. Boys who took medication for physical symptoms (e.g., headache, stomach-ache) were also more likely to be sexually initiated.

Older girls were more likely to report sexual initiation, as were girls who reported living in poorer neighbourhood environments. Girls reporting good communication with friends were more likely to be sexually initiated as were those who reported bullying others or being bullied themselves. Initiation was associated with more involvement with alcohol, tobacco and cannabis in the last 30 days as well as an unhealthy diet. Girls who were involved in music and drama activities were less likely to be sexually initiated.

### Age of sexual initiation

Participants who reported sexual initiation (25.7%, *n* = 619 of boys; 21.2%, *n* = 439 of girls) were categorised into two groups based on age of initiation; those who had initiated between age 14 and 15 (early sexual initiation, 14–15 years) and those and those who had initiated before age 14 (very early sexual initiation, < 14 years). Very early initiation was reported by 22.8% of the sexually initiated boys and 13.4% of the sexually initiated girls (Table 1).

Table 3 details the Chi^2^ tests of association between sociodemographic characteristics and participants reporting very early sexual initiation (< 14 years) (vs early sexual initiation, 14–15 years). A significantly higher proportion of younger boys and girls reported very early sexual initiation compared to older boys and girls. Boys and girls from rural areas reported higher rates of very early initiation compared with those from urban areas. A higher propotion of boys from Traveller communities reported very sexual initiation compared to those from non-Traveller communities, however caution must be exercised when extrapolating from this finding due to small sample sizes. There were no significant associations between social class, family affluence, household composition or D/CI and early sexual initiation.

### Predictors of very early sexual initiation (< 14 years)

Table 3 also presents the adjusted odds ratios for the association between very early sexual intercourse (< 14 years) and sociodemographic and lifestyle characteristics. Boys who reported very early sexual initiation were more likely to live in rural areas, get unhealthy food from their parents and report higher alcohol and cannabis involvement. They were less likely to experience negative health symptoms but reported taking medication for psychological symptoms (e.g., difficulty getting to sleep, nervousness). Boys who reported poorer communication with their friends and who reported regularly bullying others were more likely to report very early initiation.

Girls who reported very early initiation were also more likely to live in rural areas, come from non-Traveller communities and report higher cannabis involvement. Girls who reported bullying others, and being bullied themselves were more likely to report very early initiation. Girls who reported taking medication for physical symptoms and who attended more health visits (e.g., to a doctor or dentist) were less likely to report initiation before age 14.

### Contraceptive use

Participants who reported sexual initiation (25.7%, *n* = 619 of boys; 21.2%, *n* = 439 of girls) were asked to report the method of contraception used at last intercourse, if any (Table 1). Among sexually initiated young people, around 80% of boys and girls reported using condoms at last intercourse, one fifth of boys and one quarter of girls reported using the contraceptive pill and around 14% reported using withdrawal. Approximately 10% of boys and 6% of girls reported using no method of contraception at last intercourse.

Fewer younger boys (i.e., those aged 15 years) reported using condoms at last intercourse compared to older boys (i.e. those aged 17–18 years). No other sociodemographic differences were identified in relation to condom use, apart from girls from Traveller communities reporting less condom use at last intercourse than those from non-Traveller communities (Table 4). Caution must be taken when extrapolating from these findings as the same size for sample of young people from the Traveller community is very small.

### Predictors of non-condom use

Table 4 presents the adjusted odds ratios for the association between non-condom use at last intercourse and sociodemographic and lifestyle characteristics. Older boys from high (compared to low) FAS groups were less likely to report non-condom use at last intercourse. As were boys engaged in more frequent physical activity and more health protective behaviours influenced by parenting (e.g., tooth brushing and seatbelt wearing) and who reported more frequently bullying others.

Girls from non-Traveller communities, who reported more healthy food consumption, higher quality of life and more frequently being bullied were less likely to report having sex without a condom at last intercourse. Girls who reported taking medication for physical or psychological symptoms were more likely to report having sex without a condom at last intercourse.

## Discussion

This is the first nationally representative data on the sexual health and behaviours reported by young people in Ireland. Overall, 25.7% of boys and 21.2% of girls were sexually initiated. Older age was consistently predictive of sexual initiation for both boys and girls, as were alcohol, tobacco and cannabis involvement, living in poorer neighbourhoods and good communication with friends. Involvement in music and drama was protective for both boys and girls. Very early sexual initiation (< 14 years) was reported by 22.8% of the sexually initiated boys and 13.4% of the sexually initiated girls. Very early initiation was consistently associated with rural living, cannabis involvement, bullying others and attending fewer health check-ups for both boys and girls. Unprotected sex at last intercourse was reported by 10.5% of boys and 6.8% of girls. Over three quarters of young people reported using a condom at last intercourse. Boys condom use was predicted by age, higher FAS, bullying others, physical activity and health protective behaviours. Girls’ non-condom use was predicted by taking medication for physical and psychological symptoms whereas belonging to a non-Traveller community, healthy food consumption, higher quality of life and experience of being bullied was protective.

The Irish figures for young people’s sexual initiation are lower than comparable international figures. The most recent British National Survey of Sexual Attitudes and Lifestyles (Natsal-3) found that among those aged 16–24 in the UK, 30.9% reported heterosexual intercourse before age 16 years [49]. The 2013/14 international HBSC report details the proportion of 15 year olds who report having sex. The Irish figure (27% boys, 16% girls) are lower than many other countries. For example, 26% of girls and 24% of boys (Sweden), 27% of girls and 29% of boys (Hungary), and 21% of girls and 40% of boys (Bulgaria) report sexual initiation [8]. Lower rates of sexual initiation may reflect the older age for legal intercourse in Ireland (17 years) compared to other countries, or the religious context for young people growing up in Ireland which whilst they may not be practicing Catholic, may still be *culturally* Catholic thus promoting abstinence before marriage [38]. At present, the only Irish data available for comparison comes from adults retrospective accounts; the Irish Study of Sexual Health and Relationships (ISSHR) identified that among 18–24 year olds in the sample, the median age of sexual initiation was 17 years old, whereas for those participants age 60–64 was 22/23 years old [6].

The current findings demonstrate that adolescents report condom use as the most common individual method of contraception. The 2013/14 international HBSC report also publishes data on 15 year olds who use a condom at last intercourse. From the current study, around 72% of 15 year old boys and 77% of 15 year old girls in Ireland reported condom use at last intercourse. This is higher than most of the other countries, including England, Scotland and Wales [8]. Not using condoms is a matter of considerable concern, particularly for the higher proportion of boys reporting non-contraceptive use, given the increased risk of unintended pregnancy and STIs. Young people’s contraceptive choices are often influenced by biological, psychological and social factors. They are also marred by additional complications relative to their age and stage of development. For example, not only may access and cost determine contraceptive use, but choice may be inhibited by adolescents’ lack of knowledge surrounding the ‘correct’ use of contraceptives. It is also possible that one partner will assume the other has taken responsibility for contraceptive methods. Indeed communication about contraception prior to intercourse is an important predictor of contraceptive use among adolescents [50, 51]. Kirby cites communication with partner about contraception as an antecedent of adolescent contraception [51]. Concerns surrounding confidentiality may deter contact with local health services, while embarrassment, fear of side effects and stigma associated with contraceptive use (particularly for females) may discourage contraceptive use [52, 53].

The current research identified demographic, health, socio-cultural and lifestyle characteristics consistently associated with sexual initiation. As age increases, the proportion of young people reporting initiation also increases. At 18 years old, the proportion of boys and girls who report initiating sex (37.3% and 54.5% respectively) are similar to the prevalence rates of sexual activity of the young adult population aged 18 to 20 years old in Ireland (50%) [33]. Although caution must be exercised when extrapolating from the these findings due to the small sample size of 18 year old participants, this highlights the importance of focusing on young people under the age of 18 years as they are embarking upon their sexual debut and where norms of behaviour, sexual activity and practices are established [2]. Alcohol, tobacco and cannabis involvement were associated with sexual initiation among boys and girls. Cannabis involvement was also positively associated with very early initiation for both boys and girls, and alcohol involvement for boys only. These findings are in line with international research which has linked substance use with sexual risk behaviour [54, 55] and early sexual initiation [56, 57].

Good communication with friends was associated with girls and boys sexual initiation, however poor communication with friends was associated with very early sexual initiation for boys. Bullying others was associated with very early initiation for both boys and girls, and involvement in music and drama was protective of sexual initiation for both boys and girls. The circumstances surrounding participants sexual behaviours reported are not known (i.e., whether participants wanted sexual initiation at that time). However the influence of peer support, pressure and communication may be of significant importance to the timing of sexual initiation and this warrants further investigation. Templeton et al. [58] emphasise that a key form of social learning comes from sexual scripts. These originate from adult discourses and are shared among peers. Peer perceptions have also been identified as influencers of sexual initiation and behaviour. For example if adolescents perceive that most of their peers are having sex, they are more likely to report intention to initiate, and also to initiate themselves [59]. Normalisation of sexual behaviours based on peer approval, peer pressure and a desire for social inclusion, are key aspects influencing adolescents to engage in sexual activity [58]. Adolescents living in rural areas were more likely to report very early initiation. Living in rural areas which lack accessible leisure-time resources may result in boredom and sexual intercourse [60]. Rural living may also deny young people access to sexual health advice and services when they are needed. Despite no significant association between D/CI and sexual initiation, there is complex picture for those young people who report health conditions and taking medication for health conditions, and the relationship between sexual initiation. Health protective behaviours (including engaging in physical activity and toothbrushing/seatbelt wearing) were associated with boys’ condom use at last intercourse. Possible explanations may relate to them employing health promoting behaviours across different aspects of their lives.

The proportion of young people who sexually initiate at age 18 years or younger, together with the proportion of young people who report early (at age 14 or 15 years) or very early initiation (< 14 years), when they are physically, emotionally and cognitively developing highlights that young people’s sexual behaviour is of particular relevance in the Irish context where the age of legal consent for sexual intercourse is 17 years old. Similarly, the proportion of sexually initiated young people who are not using a reliable form of contraception, thus putting themselves at increased risk of STIs and unplanned pregnancy, is of importance. When deciding whether to initiate sex, adolescents may focus on the positive rewards that sex brings (i.e., intimacy, fun, emotional attachment). Even when knowledgeable about the risks, some young people may not refrain from sex to avoid potential negative outcomes [58]. The findings highlight that sexual health is a salient public health issue for health educators, practitioners and policy makers across Ireland. The risk of unintended pregnancy carries an increased burden for young people in Ireland due to the limited options available to deal with unwanted pregnancies. Unlike most developed countries, abortion is illegal in Ireland [37]. Anyone seeking a termination must travel outside of the country to do so (with the exception of some very extreme circumstances) and must bear substantial financial costs. This can lead to a greater risk of social and psychological implications for those who experience an unplanned pregnancy in Ireland, and in particular young people who may be ill-equipped to manage these circumstances. The findings call for training of health and education professionals, knowledge and skills-based, age and stage appropriate education for young people, with the addition of young people friendly access to confidential, affordable contraception.

The findings also provide data on the sociodemographic and lifestyle behaviours that relate to young people’s sexual initiation, very early sexual initiation and non-condom use. The identification of these sociodemographic and lifestyle characteristics provides important information on risk and protective factors that relate to young people’s sexual behaviour. The findings may be used for the purpose of helping to identify young people who may be most at risk of sexual risk-taking (e.g., those living in rural areas). Similarly, the identification of amenable risk and protective factors (e.g., communication skills, bullying) may provide an opportunity for targeted intervention to reduce the inequalities that place many adolescents at an increased risk of adverse health and well-being outcomes as a result of unsafe sexual practices. For example, this study has linked adolescent sexual risk behaviours with other negative health behaviours (e.g., substance use). Although it is not possible to establish a causal relationship, the design of interventions that aim to reduce multiple risk behaviours or promote multiple protective factors may result in more positive additional health outcomes. Successful interventions specifically designed to reduce alcohol, tobacco and cannabis use may therefore also impact unsafe sexual behaviours. These associations are supported by previous research linking substance use and adolescent sexual risk behaviour [54, 55]. Jackson et al. [54] recommend a holistic approach to risk behaviour prevention that adopts the shared determinants of several risk behaviours to improve adolescent health.

The findings suggest that boys and girls sexual behaviour is related to different socio-demographic and lifestyle characteristics. The differences highlight the need to incorporate gender into health promotion and behavioural education, skills, training and interventions. This may be especially important in Ireland where at post primary level (12–18 years) 63% of schools attended by 66% of pupils are single gender only. Not only will this more comprehensive, tailored approach seek to improve adolescent sexual health during this key developmental stage, but will provide a foundation for positive sexual health, behaviours and relationships throughout their life course.

The first ever sexual health strategy in Ireland was launched in 2015 [61]. The current research has the potential to inform the implementation and further development of universal sexual health promotion across settings. In terms of implementation, the findings could be employed as part of a parent education package designed to improve parent-adolescent sexual communication. They could also be used to help inform trainers preparing teachers to delivery Relationships and Sexuality Education (RSE), or in the development and delivery of similarly focused education in out of school settings. For the first time, policy-makers in Ireland have up-to-date, nationally representative data on adolescent sexual behaviour, including socio-demographic and lifestyle characteristics that are associated with risk. These and subsequent findings in the field could be used to help develop an evidence based strategy for adolescent sexual health behaviour at national, institutional (e.g., school, training centres) or community (e.g., youth services, local authority) level.

There is also potential to integrate these findings into the RSE programme implemented in secondary schools in the Republic of Ireland. RSE is delivered as part of Social, Personal and Health Education (SPHE), and SPHE is designed to be situated within a ‘whole school’ approach. Such an approach, as advocated by the Department of Education [62], requires partnerships at school level between educational stakeholders and priority setting for the school in the context of the physical and psychosocial environment. The findings reported here could be very useful to school-level partnerships in determining their approach and what knowledge and skill development to focus on for different groups of students. The early age of initiation of many young people in Ireland highlights the need to implement such interventions and education at an early age.

### Limitations

When asked about sexual intercourse, participants were provided with a description in parenthesis (Sometimes this is called “making love”, “having sex” or “going all the way”). Sexual intercourse was not however described using an anatomical definition. Neither does it differentiate between consensual or non-consensual sex. Although early sexual intercourse is often correlated with other indices, the cross-sectional design means that causality cannot be established. Similarly, the point at which a young person sexually initiated may have occurred some years prior to completion of the survey. Measures taken during the survey, for example relating to living with both parents, refer to the time of completing the survey rather than at first intercourse. While the use of a standardised survey promotes cross country comparisons, the cultural and youth specific nuances of sexual behaviours and practices may be lost. Further participation of young people in questionnaire design may help overcome some of these challenges [63].

Constraints were imposed by the structure of the data. Gender differences warranted separate analyses for boys and girls. Significance levels were reported at *p* < .10 and *p* < .05 because of small sample size and lack of power. The small sample of early initiated participants means that analytical power may be low. Missing data were not imputed and only a complete case sample were used. Listwise deletion may also have contributed to lower analytic power. As data are most likely not missing at random (MAR), it is assumed that the results generated would be replicated if a full dataset were used; however estimates may be over- or under-estimated. The youngest participants answering questions about sexual behaviour were aged 15 years. Sexual initiation was therefore defined in relation to sexual intercourse occurring at age 15 years old or younger. Only young people older than 15 will be able to select 16 or above as an age of first intercourse. A larger proportion of sexually experienced 15-year-olds, compared to older adolescents, reported very early sexual initiation. While it is possible that more young people are initiating sex at very young ages compared to one or two years previously, it is more likely an issue of censoring, due to the age at which 15-year-olds could have initiated sex is at young and very young ages.

Completion of the questionnaire required self-report data about sexual behaviours. While every effort was made to ensure that participants completed questionnaires anonymously, individually and confidentially participants may have been unwilling to disclose such that prevalence may be underestimated [64]. Only four questions were used to ask about sexual behaviour. Some of which generated inconsistent or unfeasible responses that required recoding. The methodological challenges and solutions to these issues have been reported by Young et al. [43] Finally, at present nothing is known about the circumstances of young people’s sexual behaviour; for example, contraceptive use at first intercourse, thoughts about the timing of first intercourse, the age of partner at first intercourse or different types of contraceptive methods used (contraceptive implant, injection etc.). These questions have been added to the most recent HBSC study and for which detailed analyses are required. The data should provide more context about the timing of early initiation, coercion and associated factors such as peer pressure. Similarly, parenting processes (e.g., parent-child monitoring, sexual risk communication and parental closeness) have been identified as significant influences on adolescents’ early sexual initiation, sexual activity, and condom use [65]. HBSC Ireland do not currently ask questions relating to parental processes, however future research should take account of these findings. The questionnaire measures the health behaviours of school-going 15–18 year olds in Ireland, it therefore does not represent the views of those who are not in school. School retention rates to the end of post-primary education (Leaving Certificate, after 14 years of fulltime education) was 90.6% in 2015 [66].

## Conclusion

Sexual health is a fundamental aspect of physical and social well-being and a key component of public health [2]. Sexual health status is shaped by a range of socio-cultural, psychological, physical, cognitive, religious, legal, economic and political factors, a number of which adults, and young people have little or no control over. Sexual health is dependent on the complex interaction of a combination of these factors [3]. For the first time, a detailed analysis of the sexual behaviours and practices of young people across Ireland has been conducted. The findings highlight the prevalence of sexual initiation, age of initiation and contraceptive use among a representative sample of boys and girls aged 15–18 years. The analysis has identified sociodemographic and lifestyle characteristics associated with each of these sexual behaviours capturing a profile of both those young people who are sexually initiated and those associated with very early initiation and non-condom use.

The analyses identified predictive factors related to sexual initiation, age of initiation and contraceptive use which are specific to the lives of young people, and some which are gender-specific. Young people are a distinct group with unique influences in relation to their sexual health and behaviour. The results demonstrate the value of developing and implementing specifically targeted policy and interventions which take a holistic approach in addressing the needs of adolescents. While this study provides a novel insight into potential risk and protective factors associated with risky sexual behaviour among the adolescent population, more research is required to further understand the context of young people’s sexual behaviours; including the timing and contraceptive methods used at first intercourse, frequency of intercourse, sexual practices other than intercourse and experience of STI and unplanned pregnancy. It would be preferable if future work in this area adopted a truly ecological perspective, including more comprehensive family measures. Models on which to base this ‘ecological’ research include Bronfenbrenner’s environmental systems [67], or the socio-ecological model of health [68]. These behavioural-ecological models have been applied to sexual health research, and intervention development in the contexts of AIDS prevention [69] and young people’s risky sexual behaviour [70], many of which are summarised by Hajizade-Valokolaee [71]. A comprehensive evidence base of the factors influencing young people’s sexual behaviour will provide a foundation for the development of health promotion strategies aimed at reducing negative sexual health outcomes and promoting positive outcomes. These recommendations are directly relevant to the current Irish national policy on sexual health [61], and the setting of priorities in its implementation.

## Additional file

Additional file 1: Factors derived from the Categorical Principle Components Analysis (CatPCA) conducted on individual items in each of the following four candidate domains (including proportion of variance explained (%)). Description of data: The additional file presents the factors derived from the Categorical Principle Components Analysis (CatPCA) conducted on the individual items which comprise each of the four selected candidate domains. The data presents the factors, and includes the individual and cumulative proportion of variance explained (%). (DOCX 24 kb)

## Abbreviations

FAS: Family Affluence Scale
SES: Socioeconomic status
STIs: Sexually transmitted infections
WHO: World Health Organization
