# Supplementary material for: Sexual intercourse, age of initiation and contraception among adolescents in Ireland: findings from the Health Behaviour in School-aged Children (HBSC) Ireland study
Source: BMC Public Health. 2018 Mar 16;18:362. doi: 10.1186/s12889-018-5217-z (PMC5857110; doi:10.1186/s12889-018-5217-z)
Supplement: Supplementary file 1 — Factors derived from the Categorical Principle Components Analysis (CatPCA) conducted on individual items in each of the following four candidate domains (including proportion of variance explained (%)). Description of data: The additional file presents the factors derived from the Categorical Principle Components Analysis (CatPCA) conducted on the individual items which comprise each of the four selected candidate domains. The data presents the factors, and includes the individual and cumulative proportion of variance explained (%). (DOCX 24 kb) [file 12889_2018_5217_MOESM1_ESM.docx]

**Additional file:** **Factors derived from the Categorical Principle Components Analysis (CatPCA) conducted on individual items in each of the following four candidate domains (including proportion of variance explained (%))**

***Domain 1: Positive lifestyle behaviours (cumulative variance explained 66.1%)***

Factor 1: Frequency of physical activity (20.0%)

‘Over the past 7 days, on how many days were you physically active for a total of at least 60 minutes per day?’

‘Outside of school hours: How often do you usually exercise in your free time so much so that you get out of breath or sweat?’

‘Outside of school hours: How many hours a week do you usually exercise in your free time so much so that you get out of breath or sweat?’

Factor 2: Active travel (14.6%)

‘On a typical day is the main part of your journey to school made by…?’

‘On a typical day is the main part of your journey from school made by…?’

Factor 3: Eating breakfast (12.6%)

‘How often do you usually have breakfast (more than a glass of milk or fruit juice)?’

Factor 4: Healthy food consumption: (9.8%)

‘How many days a week do you usually eat or drink….? Fruits/ vegetables/ fish’

Factor 5: Health protective behaviour: (9.0%)

‘How often do you brush your teeth?’

‘How often do you wear a seatbelt when you sit in a car**?’**

**Domain 2: Negative lifestyle behaviours (cumulative variance explained 58.2%)**

Factor 1: Alcohol involvement (31.6%)

‘At present how often do you drink anything alcoholic, such as beer, wine or spirits? Beer/wine/spirits-liquor/alcopops/cider/other’

‘Have you ever had so much alcohol that you were really drunk?’

‘Think back again over the last 30 days. How many times (if any) have you had five or more drinks in a row?’

‘At what age did you first do the following things? Get drunk’

‘On how many occasions (if any) have you done the following things in the last 30 days? Drunk alcohol/ been drunk’

Factor 2: Cannabis involvement (10.2%)

‘Have you ever taken cannabis (hashish, grass, pot)? In your life/ in the last 12 months/ in the last 30 days’

‘At what age did you first do the following things? Take cannabis’

Factor 3: Unhealthy food from parents (7.0%)

‘Do you get the following items from your parents if you ask them? Coke or other drinks that contain sugar/sweets or chocolates/biscuits or pastries/crisps’

Factor 4: Smoking involvement (5.3%)

‘How often do you smoke tobacco at present?’

‘How frequently have you smoked cigarettes during the last 30 days?’

‘On how many occasions (if any) have you done the following things in the last 30 days? Smoked cigarettes’

Factor 5: Unhealthy food consumption (4.2%)

‘How many days a week do you usually eat or drink…. Sweets/Coke or other soft drinks that contain sugar/diet coke or soft drinks/crisps/chips-fried potatoes’

**Domain 3: Health (cumulative variance explained 51.45%)**

Factor 1: Experience of health symptoms (27.6%)

‘In the last 6 months: how often have you had the following…? Headache/ stomach-ache/ back ache/ feeling low/ irritability or bad temper/ feeling nervous/ difficulties getting to sleep/ feeling dizzy’

Factor 2: Quality of life (10.0%)

‘Would you say your health is … Excellent/good/fair/poor?’

‘In general how do you feel about your life at present?’

‘In general, where on the ladder do you feel you stand at the moment? (Life satisfaction)’

‘Thinking about the last week….have you been happy with the way you are?’

Factor 3: Medication for physical symptoms (7.6%)

‘During the last month have you taken medicine or tablets for the following: Headache/ stomach-ache’

Factor 4: Medication for psychological symptoms (6.2%)

‘During the last month have you taken medicine or tablets for the following: Difficulties getting to sleep/ nervousness’

**Domain 4: Socio-cultural environment (cumulative 52.4%)**

Factor 1: Communication with friends (11.7%)

‘How easy is it for you to talk to the following person's about things that really bother you?’ ‘Friends of the same sex/ best friend/ friends of the opposite sex’

Factor 2: Sense of community (7.7%)

‘You can trust people around here’

‘I could ask for help or a favour from neighbours’

‘It is safe for young children to play outside during the day’

‘Do you think the area in which you live is a good place to live?

Factor 3: Neighbourhood environment (6.5%)

‘In the area where you live are there…? Groups of people who cause trouble/litter, broken glass or rubbish lying around/ run-down houses or buildings’

Factor 4: Bullying others (6.1%)

‘How often have you taken part in bullying another student(s) at school in the past couple of months?’

‘How often have you taken part in bullying another student(s) outside school in the past couple of months?’

Factor 5: Music and drama (4.9%)

‘How often are you involved in the following? Drama/Dance/Choir/Music/Singing classes’

Factor 6: Being bullied (4.3%)

‘How often have you been bullied at school in the past couple of months?’

How often have you been bullied outside school in the past couple of months?

Factor 7: Club or team activities (3.9%)

‘How often are you involved in the following? Sports club/team/Scouts/Guides/Youth clubs/Other clubs/groups’

Factor 8: Social interactions (3.7%)

‘How easy is it for you to talk to the following person's about things that really bother you? Mother/ father’

‘In your opinion, what does your class teacher(s) think about your school performance compared to your classmates?’

‘How well off is the area in which you live?’

‘There are good places to spend your free time (e.g. leisure centre, parks, shops)’

Factor 9: Health check ups (3.7%)

‘During the last year, how many times did you visit a family doctor?’

‘During the last year, how many times did you visit a dentist?’
